# Intrathecal Oxytocin Improves Spontaneous Behavior and Reduces Mechanical Hypersensitivity in a Rat Model of Postoperative Pain

**DOI:** 10.3389/fphar.2020.581544

**Published:** 2020-09-16

**Authors:** Antonio Espinosa De Los Monteros-Zúñiga, Guadalupe Martínez-Lorenzana, Miguel Condés-Lara, Abimael González-Hernández

**Affiliations:** Departamento de Neurobiología del Desarrollo y Neurofisiología, Instituto de Neurobiología, Universidad Nacional Autónoma de México, Queretaro, Mexico

**Keywords:** hyperalgesia, oxytocin, postoperative pain, evoked pain, spontaneous pain, allodynia, anxiety

## Abstract

The first few days post-surgery, patients experience intense pain, hypersensitivity and consequently tend to have minor locomotor activity to avoid pain. Certainly, injury to peripheral tissues produces pain and increases sensitivity to painful (hyperalgesia) and non-painful (allodynia) stimuli. In this regard, preemptive pharmacological treatments to avoid or diminish pain after surgery are relevant. Recent data suggest that the neuropeptide oxytocin when given at spinal cord level could be a molecule with potential preemptive analgesic effects, but this hypothesis has not been properly tested. Using a validated postoperative pain model (*i.e.* plantar incision), we evaluated in male Wistar rats the potential preemptive antinociceptive effects of intrathecal oxytocin administration measuring tactile hypersensitivity (across 8 days) and spontaneous motor activity (across 3 days). Hypersensitivity was evaluated using von Frey filaments, whereas spontaneous activity (total distance, vertical activity episodes, and time spent in the center of the box) was assessed in real time using a semiautomated open-field system. Under these conditions, we found that animals pretreated with spinal oxytocin before plantar incision showed a diminution of hypersensitivity and an improvement of spontaneous behavior (particularly total distance and vertical activity episodes). This report provides a basis for addressing the therapeutic relevance of oxytocin as a potential preemptive analgesic molecule.

## Introduction 

Every year, ∽240 million people undergo some type of surgery (Weiser et al., 2008; Weiser et al., 2015; Weiser et al., 2016), which causes postoperative pain and distress (*e.g.* anxiety), and in most cases impairs the patient’s quality life for a brief period (Kehlet and Dahl, 2003). Aside from postsurgical pain and hypersensitivity, patients submitted to surgical procedures experienced an increased risk (10%–50%) of developing chronic pain (Perkins and Kehlet, 2000; Chapman and Vierck, 2017). Despite the arsenal of drugs to ameliorate pain during and after these procedures (Chaparro et al., 2013; Reddi, 2016), treatments that prevent (namely preemptive analgesia) the establishment of central sensitization before the surgical incision seem to be a better strategy to ameliorate postsurgical pain (Kehlet et al., 2006; Kehlet, 2018). Certainly, as discussed by Katz and Seltzer (2009), if acute postoperative pain is reduced, so is the risk of chronic pain. This idea points to the relevance of preemptive (or perioperative) analgesia in the management of postsurgical pain.

In this context, some studies suggest that spinal oxytocin could have preemptive analgesic effects. Briefly, in preclinical experiments using female rats, Gutierrez et al. (2013) showed that after the postpartum period, endogenous spinal oxytocin release seems to diminish the hypersensitivity induced by spinal nerve ligature. Indeed, clinical data showed that the development of chronic pain in women submitted to cesarean is minor in comparison with other non-obstetric interventions (Eisenach et al., 2013). These data, coupled to fact that spinal oxytocin prevents spinal long-term potentiation (LTP) (DeLaTorre et al., 2009), a key process in the development of central sensitization leading to persistent pain (Ruscheweyh et al., 2011), point out the potential role of oxytocin as a preemptive analgesic. In the last 15 years, studies on the antinociceptive effects of spinal oxytocin have been published (for refs. See Poisbeau et al., 2018). Briefly, intrathecal (i.t.) oxytocin has been shown to reduce thermal and mechanical hyperalgesia or mechanical allodynia in neuropathic (Miranda-Cardenas et al., 2006; Sun et al., 2018; González-Hernández et al., 2019) and inflammatory pain models (Yu et al., 2003; Reeta et al., 2006). Most importantly, epidural oxytocin relieves refractory pain in humans (Condés-Lara et al., 2016).

Despite the above data suggesting the potential effect of spinal oxytocin as a preemptive analgesic molecule, this hypothesis has not been properly tested in a postoperative experimental pain condition. The present study was designed to preclinically test the preemptive antinociceptive effect of i.t. oxytocin pretreatment before a surgical procedure in a well-established model of postoperative pain. Hence, using the plantar incision model, we analyzed the effect of oxytocin on spontaneous behavior (as a surrogate model of spontaneous pain) or on evoked nociception by von Frey filaments. We found that i.t. pretreatment with oxytocin i) improves spontaneous behavior, ii) improves the recovery time, and iii) diminishes evoked hypersensitivity.

## Material and Methods

### Experimental Animals

Experiments were performed on 48 adults male Wistar rats weighing 180–220 g. The rats were provided by the bioterium of the Instituto de Neurobiología of the Universidad Nacional Autónoma de Mexico. The animals with free access to food and drinking water were housed in pairs in plastic cages with wood-based bedding, in a controlled temperature (22 ± 2°C) and humidity (50%) room under a 12:12 h light/dark cycle (light beginning at 7:00 h). For evoked pain measurements (von Frey filaments), the experiments were performed between 13:00–15:00 h; for spontaneous behavior, the experiments were performed between 17:00–09:00 h. At the end of the experiments, the animals were halted in a CO_2_ chamber.

### General Procedures

#### Plantar Incision

As previously described by Brennan et al. (1996), gaseous anesthesia with sevoflurane (4–5%; enriched with ¾ N_2_O and ¼ O_2_) was delivered with a mask and an incision of the hind paw was performed. Briefly, the plantar aspect of the left hind paw was prepared, and a 1 cm longitudinal incision was made through skin, fascia, and muscle. Under these conditions, the flexor *digitorum brevis* muscle was elevated and incised. In all cases the surgery was performed between 16:00–16:30 h. The incision started 12 mm distal from the edge of the heel and the skin was closed using 4–0 nylon sutures. Naïve rats underwent a procedure that included anesthesia and sterile preparation of the plantar area, but no incision. Immediately after surgery, the animals were divided into two main sets to evaluate the effects of oxytocin in two behavioral paradigms: i) evoked nociception induced by von Frey filaments and ii) spontaneous behavior using a semiautomated open-field system.

#### Cutaneous Evoked Nociceptive Responses (von Frey Filaments)

The von Frey filaments were used to measure the magnitude of mechanical hypersensitivity induced by the plantar incision. To assess baseline pain behavior, rats were placed individually on an elevated plastic mesh floor covered with a clear plastic cage top and allowed to acclimate. All rats were pre-tested (1 day before surgery) and tested at 1, 2, 3, 4, 5, 6, 7, and 8 days post-surgery for the response to the withdrawal threshold to von Frey filaments. Briefly, as previously described (Brennan et al., 1996), primary withdrawal to punctate stimulation was tested by applying calibrated nylon von Frey monofilaments (Touch-Test™ Sensory Evaluators, North Coast Medical, Inc., CA, USA) to an area adjacent to the incision (near the calcaneus). Each von Frey filament (14, 20, 39, 59, 78, 98, 147, 255, and 588 mN) was applied once, beginning with 14 mN until a withdrawal response occurred, if there was no paw withdrawal, 588 mN was recorded. The withdrawal threshold was calculated from the averaged of three tests.

#### Assessment of Spontaneous Activity

The rats were behaviorally tested on days 0 (basal), 1 (the same day of surgery), 2, and 3 post-surgical incision. In this case, the surgical incision was made on day 1 and the spontaneous behavior was assessed using the open-field system with arrays of photo-beam sensors to determine the location of the animal in real-time (Omnitech Electronics, Inc., OH, USA). Briefly, animals were placed individually in plexiglass boxes (16 × 16 × 11.75 inches) containing the photo-beam sensors. Locomotor activity and rearing episodes were assessed. The raw data were stored on a computer disk for off-line analyses using the Fusion v5.3 SuperFlex software (Omnitech Electronics, Inc., OH, USA). Under these conditions, spontaneous activity episodes were monitored for 16 h (starting at 17:00 h and ending at 9:00 h the following day) for 4 days. The spontaneous behavior was analyzed during the light phase (17:00–18:00 h) and the dark phase (19:00–07:00 h). In all cases we measured the horizontal activity (total distance), vertical episodes (rearing), and time (seconds) spent in the center of the open-field system. Indeed, horizontal and vertical activity have been proposed as an endpoint to indirectly analyze potential analgesic compounds (Imanaka et al., 2008; Majuta et al., 2017a), whereas time spent in the center has been used as an endpoint to analyze anxiety-related effects (File, 1980; Castanheira et al., 2018), a key issue after postoperative pain (Carr and Goudas, 1999; Kehlet and Dahl, 2003; Kouya et al., 2015).

### Drug Treatment

Under anesthesia (see above), oxytocin (10 nmol; Sigma-Aldrich, CAS: 50-56-6, base free) or isotonic saline solution (0.9% NaCl) were intrathecally (i.t.) injected in a volume of 20 µl 10 min before the plantar incision. The dose of oxytocin was chosen from previous reports showing that intrathecal oxytocin inhibits nociception in a range of 0.1–10 nmol (DeLaTorre et al., 2009; Gutierrez et al., 2013; Chow et al., 2016). The i.t. oxytocin or vehicle administration were given in a blinded manner by direct lumbar puncture (22 G) in the L5-L6 intervertebral space following the method described by Mestre et al. (1994).

### Statistical Analysis

**Figure 1:**
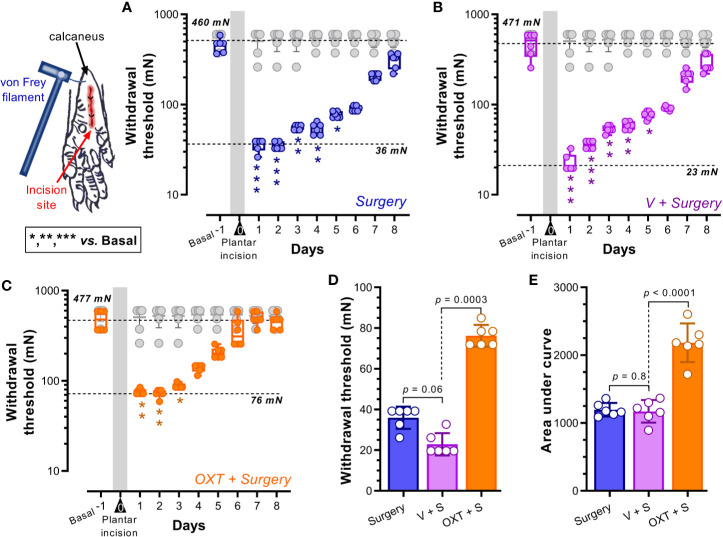
Intrathecal oxytocin (OXT) diminished the evoked pain (mechanical hypersensitivity) in a postoperative pain model. **(A–C)** show the box-plots of the primary punctate withdrawal threshold before (Basal) and after (starting 1 day after surgery for 8 days) plantar incision in animals without pretreatment (*Surgery* alone; blue) or pretreated with vehicle (*V + Surgery*; magenta) or 10 nmol OXT (*OXT + Surgery*; orange); furthermore we insert in these panels the data from naïve rats (see gray box-plot). Vehicle or OXT were given intrathecally 10 min before the plantar incision under anesthesia. The forces applied by von Frey filaments in the plantar area of the rat’s hind paw are expressed in newtons (mN). In all cases, the means of the basal withdrawal threshold were similar (≥ 460 mN), and a clear reduction of this value was induced one day after surgery (≤ 76 mN) (see the dotted lines in the panels); these data indicate that plantar incision induces mechanical hypersensitivity. Furthermore, the time necessary to find no difference in the withdrawal threshold to von Frey filaments was minor in the animals pretreated with OXT (3 days *versus* 5 days), suggesting an improvement in the recovery times. Asterisks *, **, *** indicate p < 0.01, 0.001, 0.0001 vs Basal value. **(D)** shows the means ± standard errors (SE) of the withdrawal thresholds of the different groups one day after surgery; note that the hypersensitivity in animals pretreated with OXT seems to be minor than that of *Surgery* or *V + Surgery* groups. In addition, **(E)** shows the global effect of treatments as the means of the areas under the curves (AUC) ± SE; since the AUC of animals pretreated with oxytocin is higher than that of the other two groups, we infer that oxytocin has an analgesic effect in a postoperative pain model. For statistical details, see **Table 1**.

In all cases, statistical difference was considered at p ≤0.01. The data from the von Frey filaments is represented using box and whisker plots or bar graphs [means ± standard error (SE); **Figure 1**] and were analyzed using the non-parametric Friedman test (**Figures 1A–C**) or Kruskal-Wallis test (**Figure 1D**). Furthermore, to compare the global effect of the different treatments, the area under the curve was calculated and analyzed using the parametric one-way analysis of variance (1W-ANOVA; **Figure 1E**). In the case of spontaneous activity (**Figure 3**) a Grubb’s test was initially performed to exclude outliers and we graphed the data as means ± SE. To analyze the spontaneous behavior before (basal) and after (day 1) plantar incision, we performed a paired t-test (**Figure 3A**) for each treatment. In addition, to compare the effect of different treatments for 3 days (**Figures 3B–G**), the differences were computed by the parametric two-way repeated measures analysis of variance (2W-RM-ANOVA) (in this case sphericity was not assumed). Except for the paired t-test, in all cases, to control the false discovery rate (q = 0.05) for multiple testing, we used the two-stage linear step-up procedure of Benjamini, Krieger, and Yekutieli. The statistical analysis results are detailed in **Table 1**.

**Table 1:** Statistical data with their respective *post hoc* comparison for each figure panel.

Figure	Test	*Post hoc* comparison
**1**	Friedman test for multiple comparisons	Benjamini, Krieger and Yekutieli
1A	χ^2^ = 47.31, n = 6 rats, *p* < 0.0001	Basal vs day (D): D1 (**<0.0001**); D2 (**<0.0001**); D3 (**0.0006**); D4 (**0.0004**); D5 (**0.01**); D6 (0.05)
1B	χ^2^ = 47.69, n = 6 rats, *p* < 0.0001	Basal vs day (D): D1 (**<0.0001**); D2 (**<0.0001**); D3 (**0.0002**); D4 (**0.001**); D5 (**0.01**); D6 (0.06)
1C	χ^2^ = 45.41, n = 6 rats, *p* < 0.0001	Basal vs day (D): D1 (**0.001**); D2 (**0.0003**); D3 (**0.01**); D4 (0.1); D5 (0.6); D6 (>0.99)
1	Kruskal-Wallis	Benjamini, Krieger and Yekutieli
1D	χ^2^ = 14.77, n = 6 rats, *p* < 0.0001	OXT+Surgery vs V+Surgery, p < **0.0003**
		
**1**	One-way ANOVA	Benjamini, Krieger and Yekutieli
1E	Treatment effect: F_(2, 15)_ = 50.21; p < 0.0001	OXT+Surgery vs V+Surgery, p < **0.0001**
**3**	Two-way RM ANOVA	Benjamini, Krieger and Yekutieli
3B	Interaction: F_(9, 60)_ = 2.272; p = 0.0290 Time effect: F_(1.371, 27.42)_ = 37.18; p < 0.0001 Treatment effect: F_(3, 20)_ = 8.851; p = 0.0006	Naïve vs Surgery: *p* at day 1 = **0.003**; day 2 < **0.0001**; day 3 < **0.001** Naïve vs V+Surgery: *p* at day 1 = **0.006**; day 2 = **0.0004**; day 3 = **0.002** Naïve vs OXT+Surgery: *p* at day 1 = **0.008**; day 2 = **0.0001**; day 3 = **0.002** V+Surgery vs OXT+Surgery: *p* at day 1 = 0.429; day 2 = 0.989; day 3 = 0.616
3C	Interaction: F_(9, 60)_ = 1.789; p = 0.0891 Time effect: F_(1.904, 38.02)_ = 37.18; p < 0.0001 Treatment effect: F_(3, 20)_ = 23.76; p < 0.0001	Naïve vs Surgery: *p* at day 1 = **0.001**; day 2 = **0.01**; day 3 = **0.004** Naïve vs V+Surgery: *p* at day 1 = **0.001**; day 2 = **0.009**; day 3 = **0.005** Naïve vs OXT+Surgery: *p* at day 1 = 0.02; day 2 = 0.2; day 3 = 0.3 V+Surgery vs OXT+Surgery: *p* at day 1 = **0.005**; day 2 = **0.004**; day 3 = **0.008**
3D	Interaction: F_(9, 60)_ = 3.439; p = 0.0018 Time effect: F_(2.350, 47.01)_ = 15.43; p < 0.0001 Treatment effect: F_(3, 20)_ = 9.077; p = 0.005	Naïve vs Surgery: *p* at day 1 = **0.007**; day 2 = 0.02; day 3 = 0.07 Naïve vs V+Surgery: *p* at day 1 = **0.008**; day 2 = 0.03; day 3 = 0.1 Naïve vs OXT+Surgery: *p* at day 1 = 0.02; day 2 = 0.4; day 3 = 0.09 V+Surgery vs OXT+Surgery: *p* at day 1 = 0.19; day 2 = 0.031; day 3 = **0.001**
3E	Interaction: F_(9, 60)_ = 4.778; p < 0.0001 Time effect: F_(1.930, 38.61)_ = 17.70; p < 0.0001 Treatment effect: F_(3, 20)_ = 25.14; p < 0.0001	Naïve vs Surgery: *p* at day 1 = **0.009**; day 2 = **0.002**; day 3 = **0.006** Naïve vs V+Surgery: *p* at day 1 = **0.01**; day 2 = **0.002**; day 3 = **0.006** Naïve vs OXT+Surgery: *p* at day 1 = 0.05; day 2 = 0.2; day 3 = 0.1 V+Surgery vs OXT+Surgery: *p* at day 1 = **0.01**; day 2 < **0.0001**; day 3 = **0.002**
3F	Interaction: F_(9, 60)_ = 1.85; p = 0.07 Time effect: F_(2.77, 55.40)_ = 2.58; p = 0.067 Treatment effect: F_(3, 20)_ = 1.32; p = 0.29	Naïve vs Surgery: *p* at day 1 = 0.224; day 2 = 0.225; day 3 = 0.135 Naïve vs V+Surgery: *p* at day 1 = 0.206; day 2 = 0.221; day 3 = 0.175 Naïve vs OXT+Surgery: *p* at day 1 = 0.393; day 2 = 0.789; day 3 = 0.246 V+Surgery vs OXT+Surgery: *p* at day 1 = 0.25; day 2 = 0.17; day 3 = 0.60
3G	Interaction: F_(9, 60)_ = 1.073; p = 0.396 Time effect: F_(2.324, 46.48)_ = 0.864; p = 0.442 Treatment effect: F_(3, 20)_ = 8.137; p = 0.001	Naïve vs Surgery: *p* at day 1 = 0.394; day 2 = 0.326; day 3 = 0.752 Naïve vs V+Surgery: *p* at day 1 = 0.647; day 2 = 0.631; day 3 = 0.234 Naïve vs OXT+Surgery: *p* at day 1 = **0.01**; day 2 = 0.06; day 3 = 0.02 V+Surgery vs OXT+Surgery: *p* at day 1 = **0.01**; day 2 = 0.02; day 3 = **0.003**

In all cases we considered p ≤ 0.01 (underline and bold) for significance.

## Results

### The Postoperative Pain Model and Evoked Pain

Postoperative pain was induced in animals by plantar incision of the hind paw. **Figure 1A** shows the primary mechanical withdrawal threshold before (basal; 460 mN) and after plantar incision (for 8 days); note that one day after surgery the withdrawal threshold diminished (36 mN) (p < 0.0001). This hypersensitivity to von Frey filaments remains significant until day 5 post-surgery (p = 0.01). Similar results were obtained in animals pretreated with vehicle (**Figure 1B**). In contrast, as **Figure 1C** shows, when the animals received oxytocin (10 nmol, i.t.), the hypersensitivity to von Frey filaments remained significant until day 3 post-surgery (p = 0.01). Note that the withdrawal threshold of naïve animals was not modified during the test days. Furthermore, when comparing the withdrawal threshold of animals pretreated with oxytocin *versus* untreated animals 1 day after surgery (**Figure 1D**), we found that the withdrawal threshold of oxytocin-treated rats was higher than that in untreated rats; this result implies a preemptive antinociceptive effect. Similarly, upon analyzing the global effects as area under the curve (AUC) (**Figure 1E**), we found that oxytocin pretreatment increases the AUC, suggesting that this neuropeptide decreases the evoked hypersensitivity.

### Postoperative Pain Model and Spontaneous Behavior

**Figure 2:**
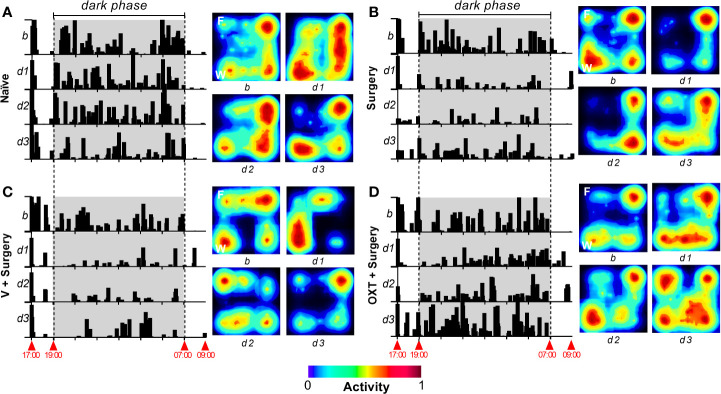
Appearance of ambulatory activity and representative heat maps of different treatments before and after the surgical procedure. In all panels, to the left, a raster plot of the ambulatory activity is shown; the data was obtained through a 16-h recording between 17:00–09:00 h for 4 days, where *b* represents the basal value, and *d1*, *d2*, and *d3* represent day 1, day 2, and day 3, respectively, after the incision of the hind paw. To the right of each panel, a representative heat map of horizontal locomotor activity is depicted, where F and W represent the site in the open-field system where the food and water were presented. In the case of naïve animals **(A)**, the actograms and heat map are similar across test days; note that, according to the heat maps, naïve animals move around the open-field system in a similar pattern across all test days. Comparable results were obtained in basal condition in all animal groups. When the animals were subjected to an incision of the hind paw [**(B–D)**], activity was clearly impaired at *d1*. Furthermore, at *d3* the spontaneous activity was partially recovered (compared to basal condition) in the *Surgery* and *Vehicle + Surgery* groups. In the case of animals pretreated with 10 nmol oxytocin (OXT) **(D)**, a minimal impact in the activity was recorded at *d1*, and a complete recovery in the activity was achieved at *d3*.

**Figure 2** shows the actograms and representative heat map of locomotor activity from naïve (**Figure 2A**), surgery untreated (**Figure 2B**), vehicle + surgery (**Figure 2C**) and oxytocin + surgery (**Figure 2D**) rats obtained during the 16 h of recording before (basal activity, B) and after (day 1, 2, and 3) surgery. As shown in the actograms, when the animals were first placed in the activity boxes during the light phase (at 17:00 h), they actively explored the novel environment; after this initial exploration, the animals remained with low activity (between 18:00–19:00 h) until the start of the dark phase (lights off at 19:00 h). In the naïve group (**Figure 2A**), an increase in locomotor activity during the dark phase was clearly observed; this spontaneous activity diminished after surgery in the surgery untreated (**Figure 2B**) and surgery + vehicle (**Figure 2C**) groups. Indeed, a visual reduction in the intensity of actograms and heat maps was achieved after surgical incision (see subpanels *d1*). In contrast, the animals pretreated with oxytocin (**Figure 2D**) showed a minor impairment of the spontaneous locomotor activity; in fact, at day 3 post-surgery, the locomotor activity and heat map were similar to the basal response.

**Figure 3:**
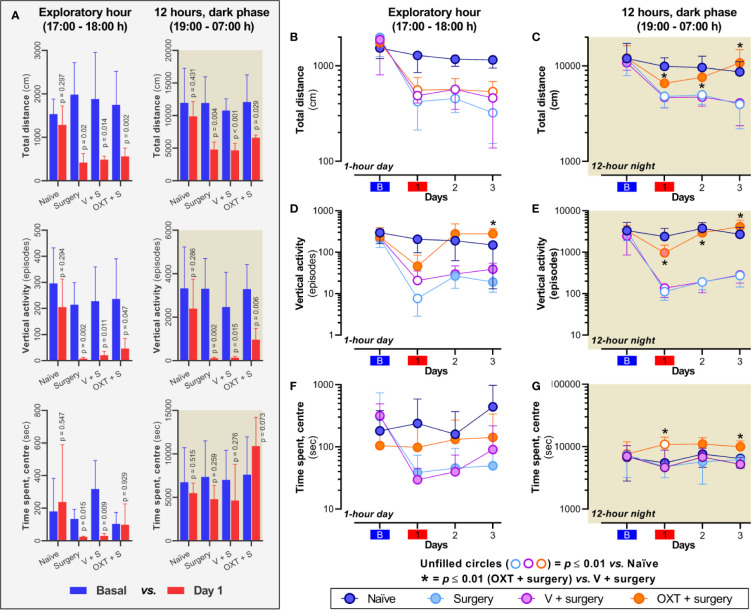
Intrathecal oxytocin (OXT; 10 nmol) consistently improves spontaneous behavior during the dark phase. **(A)** depicts the data of spontaneous behavior before (basal) and after plantar incision in the different experimental groups; note that plantar incision impairs the *total distance traveled* and *vertical activity* in all groups, and the *time spent in the center* only was impaired during the exploratory hour in surgery and vehicle + surgery animals. **(B–G)** show the changes in *total distance*, *vertical activity*, and *time spent in the center* across four days in naïve rats and rats with: i) surgery (light blue circle); ii) vehicle (V) + surgery (magenta circle); and iii), OXT + surgery (orange circle). In surgery and V + surgery groups, incision of the hind paw induces a marked decline in the total distance [**(B, C)**] and vertical rearing activity [**(D, E)**] during the exploratory and 12-h dark phase when the rats are most active. These declines peak at day 1 in the exploratory hour and remained throughout the post-surgery test days in the dark phase. Furthermore, although no effect was observed in the group of animals that were intrathecally administered with 10 nmol OXT regarding total distance during the exploratory hour, a clear improvement in this measure was induced in the dark phase; similar results were obtained upon measuring vertical activity. Panel **(F)** showed that although a diminution in the time spent in the center was achieved after surgery in all animals, no statical difference was observed. Nevertheless, as shown in panel **(G)** an slight increase in the time spent in the centre was observed in animals treated with oxytocin. In these panels, unfilled circles represent a significant difference (p < 0.01) between “OXT + surgery” vs “naïve” animals, whereas (*) represent a p < 0.01 between “OXT + surgery” vs “V + surgery”. For statistical details, see **Table 1**.

As shown in **Figure 3A**, after plantar incision, the total distance traveled and vertical activity tend to diminish in the surgery, vehicle + surgery and oxytocin + surgery groups; this effect is not observed in the naïve animals (p > 0.2). Although similar results were obtained in the time spent in the center during the exploratory hour for naïve, surgery and vehicle + surgery groups, no difference was observed before and after oxytocin treatment (p = 0.929). Also, during the dark phase the probability of finding a difference in the time spent in the center before and after surgery was low in all groups (p > 0.2); nevertheless, the rats receiving oxytocin tended to spend more time in the center (p = 0.073).

When we analyzed the impact of oxytocin treatment in comparison with the other groups, we found that after plantar incision (day 1), the *total distance traveled* diminished for the first hour of exploratory behavior and for the 12 h of nighttime activity (**Figures 3B, C**). This impairment was observed throughout the 3 days in the case of surgery and vehicle + surgery group. In contrast, in animals pre-treated with oxytocin, the activity during the dark phase was not statistically different from that of the naïve group (**Figure 3C**; p ≥ 0.02), even though after surgery a decrease in the distance traveled was found during the first hour of exploratory behavior throughout the 3 days (**Figure 3B**; p ≤ 0.008).

In the case of *vertical activity* (**Figures 3D, E**), at day 1 during light and dark phase, the number of rearing episodes (where the rat stands on its hind legs) was diminished (p ≤ 0.01) only in the groups with surgery or vehicle + surgery but not in the animals treated with oxytocin (p ≥ 0.02). Furthermore, during the dark phase, the vertical activity of the animals without oxytocin was clearly impaired on days 2 and 3 (p ≤ 0.006); interestingly, during the light phase of these days we did not find any statistical differences (p ≥ 0.02) despite the diminution of vertical activity. In animals treated with oxytocin, the number of vertical episodes displayed on days 2 and 3 was similar to that of the naïve group during the light and dark phase (p ≥ 0.09).

Finally, in the case of time spent in the center (**Figures 3F, G**), the probability that oxytocin affected this variable in comparison with the surgery untreated animals was low on the test days during the exploratory hour (p > 0.2). However, during the dark phase, the animals treated with oxytocin tended to spend more time in the center of the open field, particularly on days 1 and 3 post-surgery.

## Discussion

Using a surrogate model of postoperative pain, this study showed that spinal oxytocin pretreatment induces not only a diminution in the evoked mechanical hypersensitivity (**Figure 1**) but also an improvement of spontaneous activity (**Figures 2**, **3**). Apart from the implications discussed below, these data support our contention that oxytocin could have a potential effect as a preemptive analgesic drug. Furthermore, despite studies about the antinociceptive effect of spinal oxytocin, with perspectives ranging from neuropathic to inflammatory and from behavioral to electrophysiological (see Poisbeau et al., 2018 for refs.), we must acknowledge that no study has been performed using a postoperative pain model. This is a key point considering that each paradigm tested in previous reports has specific outcomes that are not interchangeable (Deuis et al., 2017). Specifically, postsurgical pain involves lesion of the peripheral tissue, inflammatory process and injury of isolated nerves; hence, the pain induced by surgery is considered an entity apart from other types of pain (Brennan, 2011; Reddi, 2016).

As previously demonstrated (Brennan et al., 1996; Pogatzki et al., 2002; Pogatzki and Raja, 2003), paw incision has a remarkable effect on the punctate mechanical induced-hypersensitivity, an effect associated with the sensitization of spinal dorsal horn cells in response to the spontaneous activity of Aδ- and C-fibers (Vandermeulen and Brennan, 2000; Hämäläinen et al., 2002). Indeed, as shown in **Figure 1**, allodynia towards mechanical stimuli occurred after plantar incision, but in animals pretreated with oxytocin (see **Figures 1C, D**), the impact on the withdrawal threshold on day 1 after surgery was minor, suggesting a preemptive analgesic effect. Specifically, the withdrawal threshold of animals pretreated with this neuropeptide (**Figure 1C**) was non-statistically different (p = 0.1) to the basal value after four days, whereas the threshold value of untreated animals was not statistically different until day 6 post-surgery (**Figures 1A, B**; p > 0.05), suggesting that apart from the protective effect observed on day 1, oxytocin also decreases the recovery time. These data correlate with a previous electrophysiological report showing that spinal oxytocin prevents the spinal LTP (DeLaTorre et al., 2009), one of the mechanisms whereby acute pain (in this case skin incision) induces central sensitization and consequently long-term hypersensitivity (Ruscheweyh et al., 2011). We need to mention that the spinal LTP was measured as an increase in the activity of nociceptive Aδ- and C-fibers. Hence, the electrophysiological data about the preemptive antinociceptive effect of oxytocin was translatable to our behavioral experiments measuring mechanical hypersensitivity.

Certainly, to measure allodynia that reflects a better clinical condition, we used the von Frey filaments considering that after surgery, non-noxious stimuli could be the main source of evoked pain (Jensen and Finnerup, 2014). Nevertheless, although evoked nociceptive tests are useful to evaluate potential analgesics drugs, the measurement of spontaneous behavior is crucial because provides insight into the pain process under “normal” environmental conditions (Matson et al., 2007; Gould et al., 2009; Urban et al., 2011; Cho et al., 2013; Majuta et al., 2017b; Castel et al., 2018). In humans, spontaneous pain can be quantified by asking them (Gaston-Johansson et al., 1990), whereas in rodents the locomotor activity can be analyzed as a surrogate model of this outcome (Roughan et al., 2009; Majuta et al., 2018). In addition, the classical stimulus-evoked pain-like behaviors are mainly performed during the light phase of the day, which is when rodents are less active (Yasenkov and Deboer, 2012; Frank et al., 2017).

In this sense, using an automated system we measured locomotor activity in real time for 16 h, from 17:00 to 09:00 h. When the animals were placed in the activity boxes during the light phase (17:00 h), they actively explored the new environment for approximately 1 h. After initial exploration, most of the animals remained at rest or markedly reduced their activity until the beginning of the dark phase (19:00 h) (for examples see **Figure 2**). At this point, the animals showed a significant increase in locomotor activity during the night hours (19:00–07:00 h) which decreased once the room light was turned on again (07:00 h). The increase in activity during the day (17:00–18:00 h) is consistent with exploratory behavior in search of possible threats within a new environment (Seibenhener and Wooten, 2015), while the increase in activity immediately after the room darkened (19:00–7:00 h) is consistent with the activity related to drinking, nesting, and exploring the environment to search for food. These observations and studies from several groups (Yehuda et al., 1986; Imanaka et al., 2008; Parent et al., 2012; Majuta et al., 2017a) demonstrate that spontaneous activity is reduced when there is a lesion in the body. Certainly, as discussed by Majuta et al. (2017a, 2017b, 2018), monitoring day/night activity provides an unbiased assessment of discomfort induced by paw incision and, although the common endpoint used to evaluate behavioral hypersensitivity in rodents is mechanical punctate hypersensitivity, few human studies use skin hypersensitivity as a primary endpoint. In this sense, our data using the spontaneous behavior showed that plantar incision decreases the spontaneous behavior (**Figures 2**, **3**).

As previously reported (Brennan et al., 1996; Pogatzki et al., 2002; Pogatzki and Raja, 2003), the total distance traveled, and vertical activity were reduced after paw incision in treated and untreated animals (**Figure 3A**; superior and middle panels). However, when we compared the effect of treatments on the 3 days post-surgery, we found that oxytocin clearly improved the aforementioned variables, particularly during the dark phase (**Figures 3B–E**; see **Table 1** for statistical details). Together, these data support our contention that this neuropeptide could have preemptive analgesic properties, probably by interruption of the spinal LTP (DeLaTorre et al., 2009) elicited by the plantar incision.

Considering that a previous report (Kouya et al., 2015) suggested that after plantar incision the animals develop anxiety-like behaviors (using escape-avoidance tests), we decided to analyze the time spent in the center of the cage before and after surgery. As shown in **Figure 3A** (inferior panels), we found that during the exploratory hour, untreated surgery animals spent less time (p ≤ 0.015) in the center of the cage, but this behavior was not observed in animals treated with oxytocin (p = 0.929). Certainly, under light conditions, anxious animals tend to exhibit minimal exploratory behavior; thus, spending less time in the center is predictable (Castanheira et al., 2018). Accordingly, during the dark phase we did not find a substantial statistical effect before and after surgery, although a slight increase in this variable was observed in the oxytocin-treated animals (p = 0.073). However, when we compared the effect of treatments for 3 days post-surgery, we observed an increase in the time spent (p ≤ 0.01) in the center during the dark phase in the oxytocin-treated animals.

Collectively, these data suggest that intrathecal oxytocin seems to induce anxiolytic effects, and current evidence shows that oxytocin induces anxiolytic-like effects when given at supraspinal levels (Ring et al., 2006; Neumann and Slattery, 2016). Therefore, the question remains as to how intrathecal oxytocin induces anxiolytic effects. Although the validation of this hypothesis requires additional experiments that fall beyond the scope of the present study, we propose that intrathecal oxytocin could reach supraspinal levels using the cerebrospinal fluid dynamics, thus exerting its anxiolytic effects.

Finally, and considering that we injected oxytocin 10 min before the paw incision, we must to acknowledge that oxytocin when given at central level exerts antinociception between 10 and 90 min after injection (DeLaTorre et al., 2009; Sun et al., 2018; Taati and Tamaddonfard, 2018; González-Hernández et al., 2019). Certainly, the half-life of this neuropeptide at the central nervous system seems to be nearly 20 min (Mens et al., 1983), which is enough to engage intracellular mechanisms that, in turn, block neuronal sensitization. Although in our study we did not analyze the mechanisms involved in the preemptive effect, evidence has shown that at spinal level, oxytocin blocks the neuronal activity of nociceptive Aδ/C-fibers, an action blocked by oxytocin receptor antagonists (Miranda-Cardenas et al., 2006; Condés-Lara et al., 2009; Eliava et al., 2016). Furthermore, as reviewed by Poisbeau et al. (2018) and González-Hernández and Charlet (2018), at spinal dorsal horn level, this neuropeptide recruits mechanisms/circuits associated with a diminution of nociception, for example: i) inhibition of transient potassium current (I_A_), ii) recruitment or enhancement of GABAergic transmission, and iii) desensitization of spinal TRPV1 channels.

In summary, this study showed that i.t. oxytocin pretreatment given before the incision of the hind paw not only reduces evoked pain-like behaviors but also improves spontaneous behaviors. Taken together, these data support our contention that preoperative i.t. oxytocin administration may be an alternative to provide preemptive analgesia. Further studies focusing on the translational aspects of spinal cord delivery are needed, as well as research about the precise pharmacodynamic/pharmacokinetics aspects of oxytocin effects.

## Data Availability Statement

The raw data supporting the conclusions of this article will be made available by the authors, without undue reservation.

## Ethics Statement

The animal study was reviewed and approved by Institutional Animal Care and Use Committee at the Instituto de Neurobiología (UNAM).

## Author Contributions

AE, GM-L, MC-L, and AG-H designed the experiments. AE made a draft of the manuscript. AG-H was responsible for the overall direction of the project and for editing the manuscript. All authors contributed to the article and approved the submitted version.

## Funding

This research was financially supported by the *Programa de Apoyo a Proyectos de Investigación e Innovación Tecnológica* (PAPIIT-UNAM Mexico) under Grant agreement no. IA203119 to AG-H and IN200415 to MC-L, and by the *Fondo Sectorial de Investigación para la Educación* (CONACyT-Mexico Grant No. A1-S-23631 to AG-H).

## Conflict of Interest

The authors declare that the research was conducted in the absence of any commercial or financial relationships that could be construed as a potential conflict of interest.
